# Supplementary material for: Workforce Considerations When Building a Precision Medicine Program
Source: J Pers Med. 2022 Nov 19;12(11):1929. doi: 10.3390/jpm12111929 (PMC9692406; doi:10.3390/jpm12111929)
Supplement: Supplementary file 1 [file jpm-12-01929-s001.zip › jpm-1969728-supplementary.pdf]

**Supplemental Table S1. Referral Orders to Genetics Services by Specialty by Year**

| Specialty | Year 2013 | Year 2014 | Year 2015 | Year 2016 | Year 2017 | Year 2018 | Year 2019 | Year 2020 |
|---|---|---|---|---|---|---|---|---|
| OB/GYN/ Women's Health | 799 (18) | 916 (21) | 1433 (23) | 1756 (28) | 1919 (30) | 2160 (31) | 2721 (28) | 3159 (35) |
| Oncology | 223 (8) | 393 (10) | 593 (13) | 605 (17) | 671 (16) | 869 (17) | 918 (17) | 1039 (23) |
| Family Medicine | 119 (17) | 187 (30) | 325 (36) | 382 (40) | 541 (43) | 803 (47) | 894 (53) | 969 (55) |
| Surgery | 76 (9) | 117 (11) | 255 (11) | 339 (11) | 372 (12) | 349 (13) | 406 (18) | 428 (19) |
| Cardiology | 2 (1) | 10 (5) | 9 (5) | 45 (6) | 91 (12) | 174 (18) | 194 (25) | 300 (23) |
| Breast Clinic | 60 (1) | 138 (2) | 258 (2) | 217 (2) | 231 (3) | 143 (3) | 159 (6) | 279 (6) |
| Neurology | 5 (3) | 25 (4) | 52 (4) | 34 (5) | 93 (5) | 156 (6) | 224 (8) | 261 (10) |
| Internal Medicine | 41 (7) | 107 (8) | 187 (15) | 164 (14) | 331 (15) | 259 (13) | 283 (17) | 168 (15) |
| GI | 36 (1) | 27 (2) | 73 (2) | 99 (2) | 76 (2) | 55 (3) | 62 (6) | 81 (5) |
| Nephrology | 0 (0) | 0 (0) | 4 (2) | 1 (2) | 10 (2) | 7 (3) | 27 (4) | 74 (6) |
| Endocrinology | 11 (2) | 29 (2) | 30 (2) | 13 (2) | 34 (2) | 38 (3) | 34 (4) | 70 (8) |
| ENT | 31 (2) | 34 (3) | 31 (3) | 17 (3) | 31 (3) | 32 (4) | 31 (4) | 68 (4) |
| Other | 35 (11) | 87 (29) | 91 (31) | 98 (32) | 113 (46) | 633 (48) | 641 (61) | 621 (64) |
| Total | 1438 (80) | 2070 (127) | 3341 (149) | 3770 (164) | 4513 (191) | 5678 (209) | 6594 (251) | 7517 (273) |

This data represents the number of referral orders to clinical genetics by specialty (with number of departments within a specialty in parentheses) at Sanford Health by year from January 01, 2013 to December 31, 2020. Referral rates in 2020 may have been impacted by the pandemic. In addition, Breast Clinic referral orders did not always experience growth, but this could be due the Athena program that began in 2016 [46]. The Athena program identified high risk patients via questionnaire at time of mammogram with recommendation for genetic counseling if indicated in the mammogram report. Internal Medicine referral orders only went down when the number of departments went down which was likely impacted by staffing changes.

**Supplemental Figure S1. Timeline for the Rollout of the Sanford Chip Program**

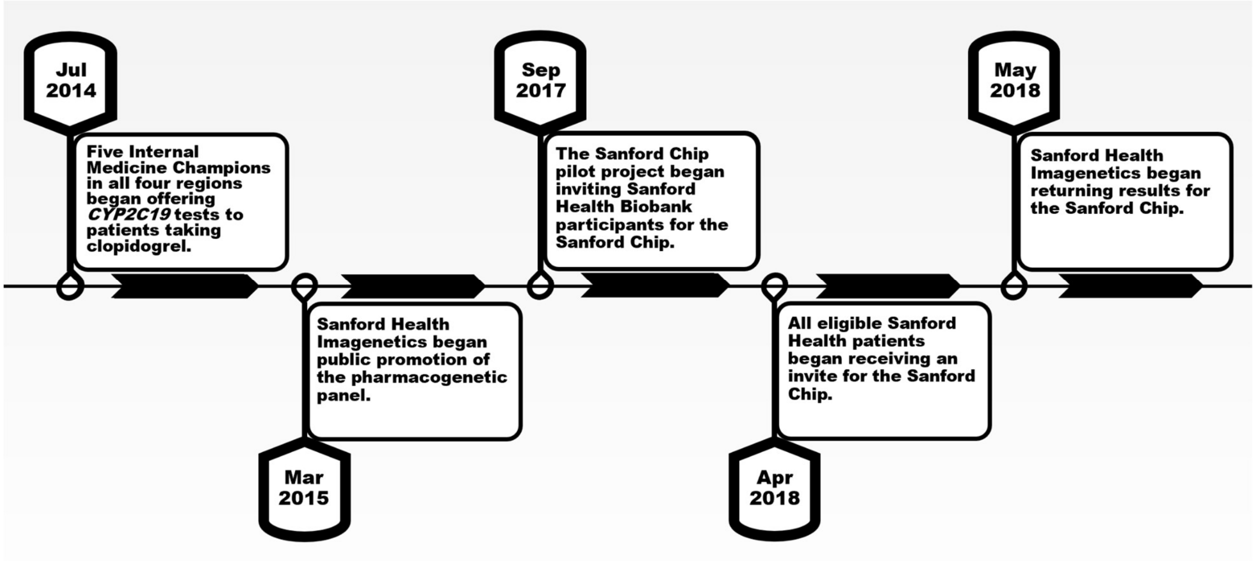


Timeline of the genetic panel creation and rollout into primary care settings across the Sanford Health system.
